# Use of tobacco and alcohol by Swiss primary care physicians: a cross-sectional survey

**DOI:** 10.1186/1471-2458-7-5

**Published:** 2007-01-12

**Authors:** Paul Sebo, Martine Bouvier Gallacchi, Catherine Goehring, Beat Künzi, Patrick A Bovier

**Affiliations:** 1 Department of community and primary care medicine, University Hospitals of Geneva, Switzerland; 2 community-based physician, Melide, Switzerland; 3 community-based physician, Biasca, Switzerland; 4 community-based physician, Bern, Switzerland

## Abstract

**Background:**

Health behaviours among doctors has been suggested to be an important marker of how harmful lifestyle behaviours are perceived. In several countries, decrease in smoking among physicians was spectacular, indicating that the hazard was well known. Historical data have shown that because of their higher socio-economical status physicians take up smoking earlier. When the dangers of smoking become better known, physicians began to give up smoking at a higher rate than the general population. For alcohol consumption, the situation is quite different: prevalence is still very high among physicians and the dangers are not so well perceived. To study the situation in Switzerland, data of a national survey were analysed to determine the prevalence of smoking and alcohol drinking among primary care physicians.

**Methods:**

2'756 randomly selected practitioners were surveyed to assess subjective mental and physical health and their determinants, including smoking and drinking behaviours. Physicians were categorised as never smokers, current smokers and former smokers, as well as non drinkers, drinkers (AUDIT-C < 4 for women and < 5 for men) and at risk drinkers (higher scores).

**Results:**

1'784 physicians (65%) responded (men 84%, mean age 51 years). Twelve percent were current smokers and 22% former smokers. Sixty six percent were drinkers and 30% at risk drinkers. Only 4% were never smokers and non drinkers. Forty eight percent of current smokers were also at risk drinkers and 16% of at risk drinkers were also current smokers. Smoking and at risk drinking were more frequent among men, middle aged physicians and physicians living alone. When compared to a random sample of the Swiss population, primary care physicians were two to three times less likely to be active smokers (12% vs. 30%), but were more likely to be drinkers (96% vs. 78%), and twice more likely to be at risk drinkers (30% vs. 15%).

**Conclusion:**

The prevalence of current smokers among Swiss primary care physicians was much lower than in the general population in Switzerland, reflecting that the hazards of smoking are well known to doctors. However, the opposite was found for alcohol use, underlining the importance of making efforts in this area to increase awareness among physicians of the dangers of alcohol consumption.

## Background

Smoking and excessive alcohol drinking affect millions of people worldwide. Health behaviours among doctors have been suggested to be an important marker of how harmful lifestyle behaviours are perceived in a country [1]. The role of tobacco in cardiovascular, pulmonary and cancer diseases has been well established [2-4]. During the last decade, many industrialised countries have seen a decrease in smoking and alcohol related cancers in men, whereas smoking related cancers continue to increase in women and in developing countries, reflecting different trends in tobacco consumption [5,6]. In several countries, the decrease of smoking among physicians was spectacular [1,7,8] and is an indication of how its hazards becomes well known in a society. Historical data have shown that because of their higher socio-economical status physicians take up smoking earlier. When the dangers of smoking become better known, physicians begin to give up smoking at a higher rate than the general population. Excessive drinking is also a cause of significant health (cirrhosis, liver cancers, chronic pancreatitis) and social problems (injuries, violence, loneliness) [9,10]. However, compared to tobacco use, many people do not perceive alcohol consumption as harmful. This is perhaps due to evidence that regular consumption of a small quantity of alcohol could protect against cardiovascular diseases [11,12]. Studies conducted in different countries have reached contradictory conclusions concerning prevalence of alcohol consumption among physicians, probably because of cultural differences and differences in risk perception [13-16].

In Switzerland, prevalence of current smoking and alcohol drinking among physicians is not known. In this paper, we used data of a previous survey [17] that included questions about tobacco and alcohol use to determine smoking and drinking behaviours among primary care physicians and compare these results with smoking and drinking behaviours of the Swiss population.

## Methods

### Sample and study design

A postal survey was conducted during the spring of 2002 among 2'756 Swiss primary care physicians identified through the membership database of the professional organisation of Swiss physicians (Federatio Medicorum Helveticorum) [17]. First, all physicians certified as general practitioners, general internists, paediatricians and physicians without a specialty qualification were identified (7'711 members). Second, a random, non stratified sample of 3'000 primary care practitioners was selected. After exclusion of 3 deceased doctors, 8 doctors with incorrect addresses, 15 doctors who did not practice clinical medicine and 218 doctors who did not practice as primary care doctors, 2'756 (91.9%) physicians remained eligible for the survey, representing 36% of all Swiss primary care physicians. The main objective of the survey was to measure the well-being of primary care physicians and its determinants. The 2'756 primary care physicians received a questionnaire with questions addressing subjective mental and physical health, burnout, medical care use, work-related satisfaction, socio-demographic and work-related characteristics, and smoking and drinking behaviours.

### Measures of lifestyle behaviours among primary care physicians

Two questions were used to measure smoking behaviour among physicians: "Do you smoke regularly (at least 1 cigarette per day)?" and "In the past, have you been a regular smoker (at least 1 cigarette per day for at least 6 months)?". Physicians were categorised as current smokers if they answered "yes" to the first question, former smokers if they answered "no" to the first question but "yes" to the second one, and never smokers if they answered "no" to both questions.

To measure alcohol use, we used the three items of the AUDIT-C score: "How often did you have a drink containing alcohol in the past year? (a "drink" to be a can or bottle of beer (2.5 dl), a glass of wine (1 dl), or one cocktail or a shot of hard liquor like scotch, gin, or vodka (0.25 dl)" (response options were never (0 points), monthly or less (1 point), 2 to 4 times a month (2 points), 2 to 3 times a week (3 points), 4 or more times a week (4 points)), "How many drinks did you have on a typical day when you were drinking in the past year?" (response optionswere 0 to 2 drinks (0 points), 3 to 4 drinks (1 point), 5 to 6 drinks (2 points), 7 to 9 drinks (3 points), 10 or more drinks (4 points)) and "How often did you have 6 or more drinks on one occasion in the past year?" (response options were never (0 points), less than monthly (1 point), monthly (2 points), weekly (3 points), daily or almost daily (4 points)). These questions have been validated for the screening of alcohol problems in men [18] and women [19]. The cut-offs of AUDIT-C score we chose in our study for the diagnosis of at-risk drinkers correspond to the optimal operating point in terms of sensitivity and specificity (≥ 5 points for men: sensitivity 91.2% and specificity 95.2%, and ≥ 4 points for women: sensitivity 81.4% and specificity 93.1%) [20,21]. Based on the answers to these 3 items, the participating physicians were categorised as non drinkers (AUDIT-C score 0), drinkers (AUDIT-C score 1 to 3 for women and 1 to 4 for men), and at risk drinkers (AUDIT-C score ≥ 4 for women and ≥ 5 for men). Distinction between at-risk consumption and dependence could not be made, because the three items of the AUDIT-C reflect only alcohol consumption [18-21]. Answers to the third question of the AUDIT-C were also used to categorise physicians as at risk drinkers (any mention of 6 drinks or more per occasion), because similar data was available for the general population (cf. below).

### Determinants of lifestyle behaviours

Socio-demographic (sex, age, living alone, economic role in the household) and work-related characteristics (medical specialty, type of practice, location of practice) were used as independent variables of smoking and drinking behaviours in an exploratory way.

### Translation of the questionnaire

The initial questionnaire was developed in French and pre-tested among a small group of physicians for readability and acceptability. When validated items in German or Italian were not available, three independent translations were made by bilingual physicians and professional translators and a final version obtained by consensus. The translated items were pre-tested before their use. The study was approved by the research ethics committee of the Institute of Social and Preventive Medicine at the University of Geneva.

### Data analysis

Prevalence of smoking and drinking behaviours among primary care physicians were calculated using frequency tables. Associations with socio-demographic and work-related determinants were tested using cross-tabulations, χ^2 ^test and linear trend test where appropriate. Agreement concerning categorization of alcohol consumers by AUDIT-C score and the third question of AUDIT-C was tested using kappa statistics. Statistical analyses were performed with SPSS (Statistical Package For Social Sciences, version 11.0).

## Results

After the first mailing and three reminders, 1784 physicians (65%) responded to the survey. The majority were men (84%) and in solo practice (63%). The mean age was 51 years (SD 8). Forty five percent were board-certified generalists, 33% general internists, 9% paediatricians and 12% practitioners without specialty qualification. Physicians who participated were younger (50.8 vs. 52.5, p < 0.001), more often men (84% vs. 78%, p < 0.001) and board-certified generalists (45% vs. 26%, p < 0.001). General practitioners had a higher response rate (72%, p < 0.001) than general internists (55%), paediatricians (65%), and physicians without board certification (41%). Over 99% of the participants responded to the items exploring smoking (n = 1'780) and drinking behaviours (n = 1'778).

### Lifestyle behaviours

Overall, 12% were current smokers and 22% former smokers. 66% were drinkers and 30% at risk drinkers according to the AUDIT-C score, respectively 62% and 34% according to the first and third question of AUDIT-C (Table 1). Only 4% were never smokers and non drinkers. Never smokers were more often non drinkers (6%) than current smokers (3%) or former smokers (2%) and non drinkers were more often never smokers (81%) than drinkers (71%) or at risk drinkers (51%). Finally, current smokers and former smokers were more often at risk drinkers (39% and 44% respectively) compared to never smokers (23%), whereas at risk drinkers were more often current smokers (16%) compared to drinkers (11%) or non drinkers (8%).

**Table 1:** Smoking and drinking behaviours among 1784 Swiss primary care physicians.

			Smoking status				Drinking behavior (AUDIT-C score)				Drinking behavior (third question of AUDIT-C)			
			Current smoker	Former smoker	Never smoker		Non drinker	Drinker	At risk drinker		Non drinker	Drinker	At risk drinker	
	n	*%*	%	%	%	*p-value*	%	%	%	*p-value*	%	%	%	*p-value*
Smoking status ^1 ^(4 missing)						*NA*				* < 0.001*				* < 0.001*
Current smoker	220	*12.4*	-	-	-		*2.8*	*49.8*	*47.5*		*2.8*	*57.8*	*39.4*	
Former smoker	398	*22.4*	-	-	-		*2.3*	*49.1*	*48.6*		*2.3*	*53.7*	*44.1*	
Never smoker	1162	*65.3*	-	-	-		*5.6*	*68.6*	*25.8*		*5.6*	*71.2*	*23.2*	
Drinking behavior ^1 ^(AUDIT-C score) (6 missing)						* < 0.001*				*NA*				*NA*
Non drinker	80	*4.5*	7.5	11.3	81.3		-	-	-		*100*			
Drinker	1166	*65.6*	10.8	18.3	70.9		-	-	-			*88.3*	*11.7*	
At risk drinker	532	*29.9*	16.2	33.0	50.8		-	-	-		-	*13.2*	*86.8*	
Drinking behavior ^1 ^(3rd question of AUDIT-C) (4 missing)						* < 0.001*				*NA*				*NA*
Non drinker	80	*4.5*	7.5	11.3	81.3		-	-	-		-	-	-	
Drinker	1102	*61.9*	9.9	17.7	72.4		-	-	-		-	-	-	
At risk drinker	598	*33.6*	17.4	32.3	50.3		-	-	-		-	-	-	

^1^: χ^2 ^test

### Characteristics associated with smoking and drinking behaviours

Smoking was more frequent among male physicians, aged 45 to 55 years, practicing in the French-speaking part of Switzerland, living alone and being the principal source of outcome in the household (Table 2). Proportion of never smokers decreased with age. At risk drinking was more frequent among men, aged 45 to 65 years, of the French-speaking part of Switzerland, living alone and being the principal contributor to the household (Table 2). Proportion of never drinkers decreased with age, then increased after 65 years. Similar trends were observed for both the AUDIT-C score and the third question of AUDIT-C, excepted for differences between geographical regions of the country and living alone. The agreement between the two measures was satisfactory (Kappa 0.76, SE 0.02) (Table 1).

**Table 2:** Socio-demographic and professional characteristics associated with smoking and drinking behaviours among 1784 Swiss primary care physicians.

			Smoking status				Drinking behavior (AUDIT-C score)				Drinking behavior (third question of AUDIT-C)			
			Current smoker	Former smoker	Never smoker		Non drinker	Drinker	At risk drinker		Non drinker	Drinker	At risk drinker	
	n	*%*	%	%	%	*p-value*	%	%	%	*p-value*	%	%	%	*p-value*
Sex^1^						*0.04*				* < 0.001*				* < 0.001*
Male	1491	*83.6*	12.6	23.3	64.0		*3.1*	*65.8*	*31.1*		*3.1*	*59.1*	*37.8*	
Female	293	*16.4*	11.0	17.5	71.6		*11.6*	*64.4*	*24.0*		*11.6*	*76.0*	*12.2*	
Age (years)^1^						*0.002*				* < 0.001*				* < 0.001*
< 35	15	*0.8*	0.0	13.3	86.7		*6.7*	*80.0*	*13.3*		*6.7*	*86.7*	*6.7*	
36–45	476	*26.7*	10.5	17.3	72.2		*5.3*	*71.6*	*23.1*		*5.3*	*66.8*	*27.9*	
45–55	829	*46.5*	14.0	22.2	63.7		*4.4*	*64.6*	*31.0*		*4.4*	*59.1*	*36.6*	
56–65	400	*22.4*	11.5	28.1	60.4		*2.3*	*59.6*	*38.1*		*2.3*	*60.0*	*37.8*	
>65	64	*3.6*	12.5	28.1	59.4		*14.3*	*66.7*	*19.0*		*14.3*	*68.3*	*17.5*	
Region^1^						*0.002*				*0.14*				*0.02*
German-speaking	1277	*71.6*	11.0	20.9	68.1		*4.2*	*67.2*	*28.6*		*4.2*	*64.1*	*31.7*	
French-speaking	437	*24.5*	16.1	26.1	57.8		*4.8*	*60.9*	*34.3*		*4.8*	*55.5*	*39.7*	
Italian-speaking	70	*3.9*	14.5	24.6	60.9		*7.1*	*65.7*	*27.1*		*7.1*	*62.9*	*30.0*	
Living alone ^1^						* < 0.001*				*0.008*				*0.08*
Yes	1665	*93.9*	26.1	15.1	58.8		*8.4*	*53.8*	*37.8*		*8.4*	*56.3*	*35.3*	
No	119	*6.7*	11.4	22.9	65.7		*4.2*	*66.4*	*29.4*		*4.2*	*62.3*	*33.5*	
Principal outcome of the household ^1 ^(13 missing)						*0.007*				*0.002*				* < 0.001*
Yes	1510	*85.3*	12.8	23.3	63.9		*4.0*	*64.5*	*31.4*		*4.0*	*59.4*	*36.6*	
No	261	*14.7*	10.0	16.2	73.8		*6.9*	*71.5*	*21.5*		*6.9*	*75.9*	*17.2*	
Medical specialty ^1^						*0.08*				*0.25*				*0.31*
General medicine	805	*45.1*	11.8	21.2	67.0		*3.7*	*66.2*	*30.0*		*3.7*	*62.1*	*34.1*	
General internal medicine	596	*33.4*	12.0	24.6	63.4		*4.0*	*65.1*	*30.9*		*4.0*	*62.0*	*34.0*	
Paediatrics	164	*9.2*	9.8	18.4	71.8		*6.7*	*68.3*	*25.0*		*6.7*	*64.0*	*29.3*	
None	219	*12.3*	17.4	23.3	59.4		*6.8*	*62.6*	*30.6*		*6.8*	*59.4*	*33.8*	
Type of practice ^1 ^(7 missing)						*0.62*				*0.45*				*0.92*
Solo	1123	*63.2*	12.3	23.0	64.7		*4.2*	*65.9*	*29.9*		*4.2*	*61.8*	*34.0*	
Group	598	*33.7*	12.4	21.6	65.9		*5.0*	*64.3*	*30.7*		*5.0*	*62.3*	*32.7*	
Other	56	*3.2*	12.5	14.3	73.2		*5.4*	*75.0*	*19.6*		*5.4*	*60.7*	*33.9*	
Place of practice ^1^						*0.18*				*0.79*				*0.86*
Urban	664	*37.3*	14.5	23.3	62.2		*4.7*	*63.9*	*31.4*		*4.7*	*60.4*	*34.9*	
Semi-urban	591	*33.2*	11.5	21.9	66.6		*4.6*	*65.8*	*29.6*		*4.6*	*62.0*	*33.4*	
Rural	524	*29.5*	10.5	21.8	67.7		*4.2*	*67.5*	*28.3*		*4.2*	*63.7*	*32.1*	

^1^: χ^2 ^test

## Discussion

Twelve percent of the primary care physicians were current smokers at the time of the survey and 30% were at risk drinkers. To our knowledge these are the first results on the prevalence of alcohol and tobacco consumption among Swiss primary care physicians, which allows for comparison with the Swiss population and other countries [1,7,8,14,22-26]. Prevalence of smoking and drinking behaviours among the Swiss general population were based on data collected by telephone during the 2002 Swiss Health Survey [27].

### Smoking behaviour

We found distinctive patterns of tobacco and alcohol consumption among primary care physicians. Primary care physicians were two to three times less likely to be current smokers when compared to the Swiss general population (12% vs. 30%).

The prevalence of smoking among physicians has decreased during the last decades in many places [8,13,28,29]. In these countries the population appears to be well informed about the dangers of smoking and physicians seem to respond by giving up smoking more frequently than other individuals, resulting in this important drop in prevalence. In several European countries and in the USA the prevalence of smoking among physicians is now lower than 20% (Table 3). When the prevalence of smoking among doctors falls effectively below that of the general population, the country's smoking epidemic is considered by some experts as "mature" [1,25,29]. By contrast, the prevalence of smoking among physicians remains very high in several southern European countries and countries with transitional economies [23,24,26,30].

**Table 3:** Smoking behaviours among medical doctors in different Western countries.

	Current smoker (%)	Former smoker (%)	Never smoker (%)
Swiss primary care physicians (2002)^7 ^(n = 1784)	12 ^1^	22	66
American cardiologist (2004)^7 ^(n = 471) [7]	2 ^1^	14	84
American physicians (1989–90)^7 ^(n = 5426) [13]	11 ^2^	40	49
Finnish general practitioners (2001)^7 ^(n = 697) [28]	13 ^3^	-	-
Estonian physicians (2002)^7 ^(n = 2668) [25]	13 ^4^	20	67
Finnish physicians (2001)^7 ^(n = 2075) [29]	15 ^3^	37	48
German physicians (1999)^7 ^(n = 1144) [8]	18 ^4^	22	60
Japanese physicians (2000)^7 ^(n = 3771) [22]	20 ^3^	28	52
Italian chest physicians (1995)^7 ^(n = 605) [30]	25 ^5^	34	41
Italian physicians (1999)^7 ^(n = 501) [24]	28 ^3^	27	46
French general practitioners (1998)^7 ^(n = 2073) [26]	32 ^6^	46	22
Bosnian general practitioners (2002)^7 ^(n = 110) [23]	40 ^5^	14	46

^1^: daily smoker.

^2^: smoking at least once in the past month.

^3^: daily or occasional smoker.

^4^: current smoker.

^5^: definition not available in the paper.

^6^: regular or occasional smoker.

^7^: year of survey.

### Drinking behaviour

A reverse pattern was found for alcohol consumption. At the time of the survey Swiss primary care physicians drank more than the general population and only 4% were abstinent (vs. 22% in the general population). In addition, those who drink consume more: 30% of the primary care physicians were classified as at risk drinkers vs. 15% for the general population.

In spite of differences in the definition used to define at risk drinker, studies conducted in other countries (Table 4) have also found high prevalence of alcohol consumption among physicians [7,13-16] Some authors suggest that the higher prevalence of alcohol use among physicians could be explained by their higher socio-economic status [13,16]. Others advocate that medical institutions, medical schools and hospitals lack efficient health promotion programs to prevent alcohol use and abuse among future and current physicians [31]. Finally, the higher use of alcohol among physicians could be directly related to the commonly accepted evidence showing that drinking a small quantity of alcohol can protect against cardiovascular diseases and is therefore not a risky behaviour for health [11,12]. Hence the results of our study point out clearly that primary care physicians not only drink more frequently, but drink more alcohol when they drink, which increases the risk of future alcohol abuse and dependence. However the different studies conducted over the last 20 years among American physicians have shown an increase of abstinence, indicating that the perception of the hazards of alcohol consumption has possibly changed among physicians in this country.

**Table 4:** Drinking behaviours among medical doctors in different Western countries.

	Drinker (%)	At risk drinker (%)	Non drinker (%)
Swiss primary care physicians (2002)^7 ^(n = 1784)	66 ^1^	30 ^1^	4
American physicians (1984)^7 ^(n = 337) [16]	86	10 ^2^	4
American physicians (1989–90)^7 ^(n = 5426) [13]	68 ^3^	9 ^3^	23
American physicians (2002)^7 ^(n = 104) [15]	66 ^4^	8 ^4^	26
American cardiologist (2004)^7 ^(n = 471) [7]	69	3 ^5^	28
Finnish physicians (1986)^7 ^(n = 2671) [14]	76	16 ^6^	8

^1^: based on the AUDIT-C score.

^2^: > 61 drinks/month.

^3^: drinker: drinking at least once during the past month; at risk drinker: ≥ 5 drinks/day at least once during the past month.

^4^: drinker: drinking ≥ 4 days/month during the past year; at risk drinker: ≥ 5 drinks at least once during the past month.

^5^: 3–4 drinks/day.

^6^: >200 g. alcohol/week.

^7^: year of survey

### Limitations

Several limitations of the current study must be kept in mind when considering the results. Firstly, the comparability with the 2002 Swiss Health Survey and international studies listed in Tables 3 and 4 may be altered for several reasons. The comparison between primary care physicians and the Swiss general population may be biased, because data had not been collected in the same manner during both surveys. Self-administered questionnaires identified by an anonymous number were used for physicians, whereas personal telephone interviews were performed for the other survey in the general population. Both methods raise the issue of underreporting and measurement error. For the physicians' survey, we believe this was unlikely, because the main aim of the study was to assess physicians' subjective health and well-being and not desirable lifestyle behaviors. In addition the questions used, especially for alcohol, were not strictly identical which raises the issue of measurement bias. In the 2002 Swiss Health Survey the questions used for smoking status were "Do you smoke?" and "Have you been a regular smoker for at least 6 months?" and for alcohol drinking "What is your usual alcohol consumption ?", "Do you drink several times during the day?" and "During the past year, how many times did you have 8 drinks (for men)/6 drinks (for women) of beer, wine, or other alcoholic beverage ?". Compared to the items we used (cf Methors section), we believe that the questions were similar enough to limit this type of error. However, several other differences may also alter the comparability between these studies, mainly the medical specialty (see Tables 3 and 4), and sexe and age variation across studies. Secondly, due to the cross-sectional nature of the survey, we cannot draw definitive causal conclusions about the observed relationships between socio-demographic characteristics and current smoking and at risk drinking. Finally, as we could not differentiate between at risk drinkers and dependant drinkers, one could argue that our results are not relevant and subject to classification bias. However, several authors consider that it is essential in term of public health to consider drinkers and at-risk drinkers, because at this stage most consequences of alcohol use are still reversible [18-21].

### Strengths

Our study included a large sample of primary care physicians and the global participation rate (65%), corresponding to 23% of the Swiss practitioners, can be considered as excellent for a postal questionnaire consisting of 16 pages. This response rate compares favourably with other similar studies performed among healthcare professionals [13,15]. Participation rate was particularly high for general practitioners, probably reflecting their high interest for this survey on their own health and well-being. Finally, the sample was also sufficiently large to allow exploration of even weak associations between variables.

## Conclusion

At the time of the survey, Swiss primary care physicians had a distinctive pattern of tobacco and alcohol consumption when compared to their compatriots. Smoking was less prevalent and alcohol drinking, particularly at risk drinking, more prevalent. If the low prevalence of current smokers reflects that the hazards of smoking is well known of primary care physicians, the reverse pattern for alcohol use underlines that efforts need to be made for this potentially harmful behaviour. Health promotion programs to limit alcohol use and abuse should not target only the general population, but also specific groups such as medical schools students, hospitals residents, and medical professional organisations to raise health professionals' awareness about the consequences of at-risk drinking.

## Competing interests

The author(s) declare that they have no competing interests.

## Authors' contributions

MBG, CG, BK and PAB participated to the planning and the conduct of the survey. PS, MBG and PAB contributed to the statistical analyses and interpretation of the results. MBG and PS reviewed the scientific literature. PS and PAB drafted the first version of the paper. All authors have read and approved the final manuscript.

## Pre-publication history

The pre-publication history for this paper can be accessed here:


